# Oral Health Assessment in Adolescents with End-Stage Chronic Kidney Disease

**DOI:** 10.3390/jcm15010165

**Published:** 2025-12-25

**Authors:** Natalia Sergeevna Morozova, Ekaterina Andreevna Maslikova, Alina Alekseevna Elovskaya, Olga Vladimirovna Nesterova, Arif Fuad Allahverdiyev, Natalya Zhorzhevna Dikopova, Alexander Grigorievich Volkov, Oleg Vitalievich Sergeyev, Ellina Valerievna Velichko, Larisa Dmitrievna Maltseva, Olga Leonidovna Morozova

**Affiliations:** 1 Department of Dental Diseases Propaedeutics, E.V. Borovsky Institute of Dentistry, Sechenov First Moscow State Medical University (Sechenov University), 117418 Moscow, Russia; morozova_n_s_2@staff.sechenov.ru (N.S.M.); arif_17_03_1998@mail.ru (A.F.A.); 2 Department of Pediatric, Preventive Dentistry and Orthodontics, E.V. Borovsky Institute of Dentistry, Sechenov First Moscow State Medical University (Sechenov University), 121059 Moscow, Russia; maslikova_e_a@staff.sechenov.ru (E.A.M.); elovskaya_a_a@staff.sechenov.ru (A.A.E.); 3 Department of Chemistry, A.P. Nelyubin Institute of Pharmacy, Sechenov First Moscow State Medical University (Sechenov University), 119571 Moscow, Russia; nesterova_o_v@staff.sechenov.ru; 4 Department of Therapeutic Dentistry, E.V. Borovsky Institute of Dentistry, Sechenov First Moscow State Medical University (Sechenov University), 121059 Moscow, Russia; dikopova_n_zh@staff.sechenov.ru (N.Z.D.); volkov_a_g@staff.sechenov.ru (A.G.V.); 5 Department of Microbiology, Virology and Immunology Named After A.A. Vorobyov, Sechenov First Moscow State Medical University (Sechenov University), 119019 Moscow, Russia; sergeev_o_v@staff.sechenov.ru; 6 Department of Pathophysiology, Institute of Digital Biodesign and Artificial Intelligence in Medicine, Sechenov First Moscow State Medical University (Sechenov University), 119019 Moscow, Russia; velichko_e_v@staff.sechenov.ru (E.V.V.);; 7 Department of Pathophysiology and Clinical Pathophysiology, Federal State Autonomous Educational Institution of Higher Education “N.I. Pirogov Russian National Research Medical University” of the Ministry of Health of the Russian Federation (Pirogov Russian National Research Medical University), 117513 Moscow, Russia

**Keywords:** chronic kidney disease, oral health, oral hygiene, microbiota, biomarkers, adolescents

## Abstract

**Background/Objectives**: End-stage chronic kidney disease (ESKD) represents a complex condition that also impacts oral health. This pilot study evaluates and compares some approaches to oral health assessment and aims to define the specific oral features common in adolescents with ESKD. **Methods**: A total of 50 children aged 12 to 17 years were examined, including 30 adolescents with ESKD (Group 1) and 20 adolescents without urinary pathology (Group 2). The decayed, missing, filled teeth (DMFT) index, oral hygiene index-simplified, papillary marginal attached index, and periodontal index were used for dental and periodontal assessment. The Milwaukee PH56 device was used to determine salivary pH. Oral microbiota was analyzed by chromatography–mass spectrometry and polymerase chain reaction detection of periodontopathogenic bacteria. Salivary and gingival crevicular fluid (GCF) biomarkers (IL-1β, TNF-α, IL-8, VEGF, sIgA) and total antioxidant capacity (TAC) were determined using an enzyme-linked immunosorbent assay. **Results**: DMFT did not differ between the groups. Periodontal indices in Group 1 were increased compared to Group 2 (*p* < 0.0001). Salivary pH in Group 1 was slightly alkaline; in Group 2, it was slightly acidic (*p* < 0.0001). Oral dysbiosis and periodontopathogenic bacteria were found in ESKD adolescents. Salivary IL-1β, TNF-α, VEGF, and IL-1β in GCF were elevated in Group 1 compared to Group 2 (*p* < 0.05). TNF-α, IL-8, and VEGF in GCF and TAC in both fluids were lower in Group 1 compared to Group 2 (*p* < 0.0001). Salivary IL-8 and sIgA in both saliva and GCF did not differ between the groups. **Conclusions**: ESKD adolescents had poor oral hygiene and significant oral dysbiosis including periodontopathogenic bacteria. Evaluation of biomarkers in saliva and GCF allowed us to vindicate inflammation, dysbiosis severity, and periodontal diseases.

## 1. Introduction

Chronic kidney disease (CKD) is characterized by abnormalities in kidney structure or function that persist for three or more months and often begins in early childhood [1]. According to the North American Pediatric Renal Transplant Cooperative Study (NAPRTCS), the main cause of CKD in children (40–60% of cases) is congenital anomalies of the kidneys and urinary tract (CAKUT) [2]. CKD affects 8% to 13% of the global population [3]. End-stage chronic kidney disease (ESKD) results from progressive loss of renal function when the host cannot offset kidney damage and requires renal replacement therapy such as dialysis or kidney transplantation [4].

ESKD is associated with multi-organ involvement along with the side effects of medications used in its treatment and can result in oral complications [5]. Dental diseases in ESKD children occur in 90% of cases [6]. The most common oral manifestations in ESKD children are dental caries, changes in oral mucosa, hyposalivation, and periodontal diseases [7].

Periodontal diseases in ESKD children and adolescents occur in up to 95.8% of cases [8]. It is a hidden, but important and often underestimated reason of general infectious complications such as bacterial endocarditis and endarteritis [9]. One of the most typical periodontal diseases in ESKD patients is drug-induced gingival overgrowth (DIGO). DIGO can be caused by a number of drugs, including immunosuppressive and antihypertensive drugs, which are mandatory before and after transplantation [10]. Adverse consequences of DIGO include difficulty in oral hygiene, ectopic teeth, impaired articulation, and unsightly gingival thickening, which may lead to emotional and social problems such as depression, anxiety, and frustration, especially in adolescents [8].

Oral diseases are associated with microbial balance disruptions, which affects the human microbiome through bacterial translocation and bacteremia. Studies of oral microbiota in CKD have been conducted primarily in adult patients [11,12]. Data in children and adolescents with ESKD are very limited. K. Höfer et al. examined the tongue microbiota in patients aged 6–25 years after transplantation and isolated patients on dialysis, but the results did not reveal significant changes in the species of microorganisms compared to control [13]. Therefore, oral microbiota in CKD children requires further studies.

Oral diseases also have bidirectional association with saliva and gingival crevicular fluid (GCF). These biological fluids contain a diverse array of components, including minerals, hormones, enzymes, immunoglobulins, cytokines, etc. The most key biomarkers are proinflammatory cytokines (IL-1β, TNF-α), chemokine (IL-8), vascular endothelial growth factor (VEGF), secretory immunoglobulin A (sIgA), and total antioxidant capacity (TAC) [14]. Interleukin-1β (IL-1β) is secreted in response to the activity of periodontopathogenic bacteria, and Interleukin-8 (IL-8) functions as a chemotactic factor, attracting neutrophils and other immune cells to the inflammation site [15]. Tumor necrosis factor alpha (TNF-α) stimulates the production of proinflammatory cytokines such as IL-1β and IL-6, which enhances the inflammatory response in oral and periodontal tissues [15]. In addition, TNF-α promotes the expression of the receptor activator of nuclear factor-κB ligand (RANKL) in periodontal ligament cells, which leads to osteoclasts activation and alveolar bone destruction [16]. Vascular endothelial growth factor (VEGF) stimulates endothelial cell proliferation, increases vascular permeability, and it is involved in angiogenesis, regulation of tissue vascularization, and hypoxia compensation [17]. Secretory immunoglobulin A (sIgA) is a key factor in the first defense line of oral immunity. Through direct interaction with microbial antigens, it maintains commensal homeostasis, thereby preventing the spread of pathogens. sIgA is synthesized by plasma cells in oral mucosa and salivary glands under the influence of proinflammatory cytokines, especially IL-1β and TNF-α, and, along with other salivary proteins and antioxidant protection, it provides a nonspecific immune response in the oral cavity [18]. The total antioxidant capacity (TAC) reflects the activity of oxidative stress and limits damage caused by reactive oxygen and nitrogen generated during the inflammation process in oral cavity [19]. Thus, analysis and profiling of biomarkers in saliva and GCF could be a non-invasive and valuable diagnostic tool for ESKD children.

The science literature contains a limited number of studies examining the relationship between oral health, dental indices, and biomarkers in ESKD children. We have found a modern article which combines oral health assessment and salivary parameters [20], but no oral microbiota findings. So, this pilot study evaluates and compares some approaches to oral health assessment: dental and periodontal assessment, chemical, microbiological, and immunological analysis; and aims to define the specific oral features common in adolescents with end-stage kidney disease.

The null hypothesis of the study was that there would be no significant difference in oral health between ESKD adolescents and healthy controls.

## 2. Materials and Methods

### 2.1. Subjects

This study involved 50 children aged 12 to 17 years, who were divided into two groups: Group 1—30 patients with ESKD (ICD-10 diagnosis codes: N18.5 Chronic kidney disease, stage 5 and T86.1 Complications of kidney transplant), Group 2—20 adolescents without urinary pathology. ESKD patients received the same immunosuppressive treatment: tacrolimus (1–9 mg/day), everolimus (2 mg/day), methylprednisolone (2–14 mg/day), and mycophenolic acid (180–720 mg/day).

### 2.2. Study Design

The pilot bicenter prospective analytical cohort study was conducted from January 2023 to June 2024. Group 1 children were examined at the Surgical Department No. 1 of V.I. Shumakov National Medical Research Center of Transplantology and Artificial Organs, and Group 2 children were examined at the Pediatric, Preventive Dentistry and Orthodontics Department of E.V. Borovsky Institute of Dentistry in Sechenov University, Moscow, Russian Federation.

This study collected demographic characteristics, including age and sex, and oral hygiene habits such as toothbrushing frequency and dental flossing using. All patients in both ESKD and control groups underwent extraoral and intraoral dental examination. The decayed, missing, filled teeth (DMFT) index, the oral hygiene index-simplified (OHI-S), papillary marginal attached (PMA) index, and periodontal index (PI) were used for dental and periodontal assessment. The Milwaukee PH56 device was used to determine salivary pH. Oral microbiota was analyzed by chromatography–mass spectrometry and polymerase chain reaction (PCR) detection of periodontopathogenic bacteria. Salivary and gingival crevicular fluid (GCF) biomarkers of inflammation (IL-1β, TNF-α, IL-8), angiogenesis (VEGF), secretory immunoglobulin A (sIgA), and total antioxidant capacity (TAC) were determined using the enzyme-linked immunosorbent assay (ELISA).

### 2.3. Ethical Review

This study was approved by the Local Ethical Committee of the Federal State Autonomous Educational Institution of Higher Education I.M. Sechenov First Moscow State Medical University of the Ministry of Health of the Russian Federation (Sechenov University) (Protocol No. 20-22 dated 20 October 2022).

### 2.4. Inclusion Criteria and Exclusion Criteria

Inclusion criteria: age from 12 to 17 years; established ICD-10 diagnosis codes: N18.5 Chronic kidney disease, stage 5, and T86.1 Complications of kidney transplant; immunosuppressive treatment including tacrolimus, everolimus, methylprednisolone, and/or mycophenolic acid; written informed consent of the patient (from 15 years old), or one of his parents, or his caregiver (for patients under 15 years old) to participate in the study according to Russian legislation.

Exclusion criteria: age under 12 and over 17 years; intercurrent forms of infectious and inflammatory diseases; sepsis; concomitant pathology (diabetes mellitus, respiratory failure, cardiovascular failure, etc.) not associated with CKD; mental disorders; refusal to participate in the study at any of its stages.

### 2.5. Oral Health Analysis

All adolescents in both groups were examined by the same pediatric dentist (E.A.M.). Dental caries was assessed using the DMFT index. Teeth were classified as decayed (D), missing (M), or filled (F). The DMFT score was calculated as the sum of D, M, and F components for each participant.

**Figure 1:**
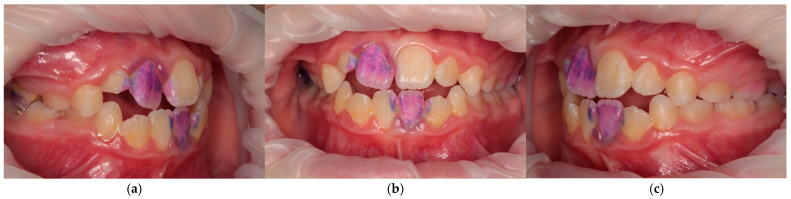
The intraoral photo protocol of a Group 1 patient. The J.C. Greene and J.R. Vermillion oral hygiene index-simplified (OHI-S) is demonstrated: (**a**) occlusion on the right; (**b**) occlusion in front; (**c**) occlusion on the left.

Periodontal assessment was performed by using the J.C. Greene and J.R. Vermillion oral hygiene index-simplified (OHI-S) to assess debris (plaque) and calculus (Figure 1); the papillary marginal attached (PMA) index assessed gingival inflammation, and the A.L. Russell Periodontal index (PI) measured periodontal disease. These evaluations were conducted using standardized measurement criteria with headlamp, dental mirror, periodontal probe, and plaque-disclosing agents. There were not any specific instructions before indexing. Children used their habitual oral hygiene technique.

OHI-S was used to evaluate oral hygiene. Dental deposits and calculus were assessed on six index teeth: vestibular surfaces of 1.1, 1.6, 2.6, 3.1 teeth and lingual surfaces of 3.6, 4.6 teeth. The OHI-S score was calculated as the sum of dental deposits and calculus scores.

Gingival inflammation was assessed by the PMA index in Parma modification. The gingiva surrounding each tooth was colored and examined separately for the papillary (P), marginal (M), and attached (A) gingival units. Each affected unit was recorded, and the PMA index was calculated as the percentage of inflamed gingival units relative to the total number of the examined teeth.

Periodontal disease was assessed by PI. The periodontal tissue around each tooth was probed and scored according to the presence of gingival inflammation and its severity, periodontal pocket formation, and tooth mobility, using A.L. Russell’s criteria. PI scores were calculated as the mean score of all examined teeth.

### 2.6. Chemical Analysis

**Figure 2:**
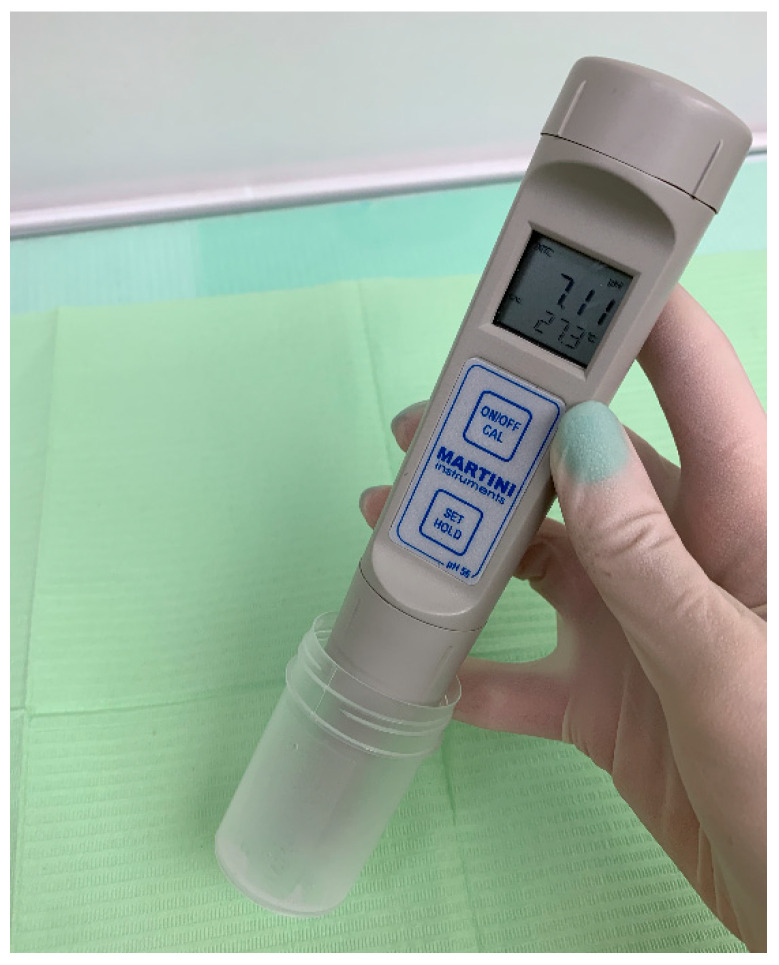
The Milwaukee PH56 professional acidity analyzer (Milwaukee Electronics, Inc., Milwaukee, WI, USA).

The Milwaukee PH56 portable acid–base balance meter was used to determine salivary pH (Figure 2). Each participant was provided with a disposable cup and instructed to collect unstimulated saliva by passive drooling over a period of 5–15 min. After sample collection, the activated Milwaukee PH56 device was immersed with its electrode into the sample, and salivary pH value was recorded.

### 2.7. Microbiological Analysis

Microbiological analysis was performed in the morning before breakfast, toothbrushing, and medication intake. Oral mucosa samples were scraped by sterile universal probe type A and microbial molecular markers were detected by G.A. Osipov chromatography–mass spectrometry—the method based on molecular indication of microorganisms in the analyzed sample [21]. Then, each analysis was assessed using classification by V.V. Khazanova et al. [22]. According to this classification, the severity of dysbiosis was evaluated using a five-grade scale, where

- Grade 1 corresponded to a normal state of oral microbiota;
- Grade 2 indicated a dysbiotic shift characterized by the predominance of a single opportunistic microorganism with the preservation of normal microbial species in oral microbiota;
- Grade 3 (grade I–II dysbiosis) represented a subcompensated form with more pronounced alterations in microbial composition, including two or three pathogenic species accompanied by a partial reduction in normal microbiota;
- Grade 4 (grade II–III dysbiosis) was defined by the detection of a pathogenic monoculture with a marked decrease or complete absence of representatives of the normal microbiota;
- Grade 5 (grade IV dysbiosis) was characterized by the presence of pathogenic bacterial associations with a marked increase in yeast-like fungi.

Periodontal pathogens were detected using polymerase chain reaction (PCR) analysis. Sampling was performed in the morning under fasting conditions and prior to toothbrushing. Sterile absorbent paper points (ISO 0.02, sizes 25–40, Gapadent Co., Tianjin, China) were inserted into the gingival sulcus for gingival crevicular fluid (GCF) absorption. The paper points were placed into Eppendorf tubes containing the “DNA-EXPRESS” reagent. Then samples were transported to the laboratory in a thermal container for PCR detection of periodontopathogenic bacteria. Color-coding of the bacterial groups was based on their association with microbial complexes by Socransky et al. [23]: “red complex”—*Tannerella forsythia*, *Treponema denticola, Porphyromonas gingivalis*; “orange complex”—*Fusobacterium nucleatum*, *Prevotella intermedia*; “green complex”—*Aggregatibacter actinomycetemcomitans*.

### 2.8. Immunological Analysis

Saliva and GCF samples were collected by the absorption method in the morning before breakfast, toothbrushing, dialysis session, and immunosuppressive drug intake. Salivary and GCF biomarkers of inflammation (IL-1β, TNF-α, IL-8), angiogenesis (VEGF), secretory immunoglobulin A (sIgA), and total antioxidant capacity (TAC) were measured by enzyme-linked immunosorbent assay (ELISA) using Vector-Best reagent kits (Novosibirsk, Russia). All specimens were measured in duplicate wells, and the mean of two values was taken. The detection limits of IL-1β, TNF-α, IL-8, and VEGF were 1.0, 1.0, 2.0, and 10 pg/mL, respectively. The detection limit of sIgA was 0.35 mg/L and of TAC—0.25 mmol/L.

### 2.9. Statistical Analysis

A Shapiro–Wilk test was applied to assess the normality of data distribution. Homogeneity of variances assumptions were examined using the Levene test. Power analysis determined the sample size and effect size (Cohen’s d). Sample size calculations ensured 95% statistical power at α = 0.05. The Kruskal–Wallis test was used for variables that failed the normality or homogeneity of variance tests.

Student’s *t*-test was used for normally distributed data, Welch’s *t*-test was used for normally distributed data with unequal variances, and the Mann–Whitney U test was used for non-normally distributed data. Differences between groups were assessed using Welch’s *t*-test for continuous variables and Fisher’s exact test for categorical variables. Statistical significance was determined for values of *p* < 0.05. To account for multiple comparisons, *p*-values were adjusted using the Bonferroni correction. The results were processed using GraphPad Prism software, version 8.0.1.

Sex, oral hygiene habits (toothbrushing frequency and dental floss use), grades of dysbiosis, and frequency of periodontopathogenic bacteria occurrence were analyzed as categorical variables, presented as absolute and relative frequencies, and compared between groups using Fisher’s exact test. Age, indices, pH, salivary TNF-α, IL-8, sIgA, TAC, and all biomarkers in GCF were treated as normally distributed; salivary IL-1β and VEGF were treated as non-normally distributed variables.

The data of biomarkers were presented as box-and-whiskers plots. The boxes span from the 25th to the 75th percentile, the whiskers span from the lowest to the highest observations, and the line inside each box denotes the median. Statistically significant differences between the groups were considered at *p* < 0.0001—** and *p* < 0.05—*.

Correlation analysis quantified the strength and direction of associations between parameters. Spearman rank correlation examined the relationship between salivary markers and microbial composition characterized as oral dysbiosis degree. The Pearson correlation coefficient measured the relationship between GCF biomarkers and the presence of periodontopathogenic bacteria. Color-coded gradations characterized *r*—strength of the identified correlation, where green corresponded to direct correlation and red to inverse correlation.

## 3. Results

This study was conducted on a sample of 50 children aged between 12 and 17 years. The mean age of participants was 13.4 ± 1.8 years in Group 1 and 13.7 ± 1.9 years in Group 2, with girls accounting for 30% and 20%, respectively. Toothbrushing once daily was reported by 63.3% of participants in Group 1 and 65.0% in Group 2, while brushing twice or more per day was reported by 36.7% and 35.0%, respectively (*p* = 1.000). The use of dental floss was reported by 10.0% of participants in both groups, whereas the majority of participants did not use dental floss (90.0% in each group) (*p* = 1.000). No significant differences were found between participants. The baseline characteristics of the study groups are presented in Table 1.

### 3.1. Oral Health Status

Oral health assessment is shown in Table 2. Dental examinations for both groups showed that some adolescents had no caries or fillings, whereas others had more than five cavities and some missed teeth. Group 1’s DMFT averaged 1.27 ± 2.12 and Group 2’s DMFT averaged 1.6 ± 1.45, so it did not demonstrate statistically significant differences between the groups (*p* > 0.05).

**Figure 3:**
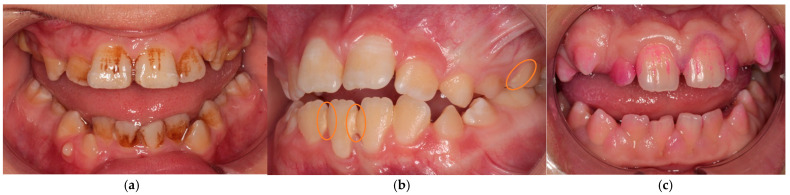
The intraoral photo protocol of Group 1 patients: (**a**) dense pigmented plaque; (**b**) dental calculus; (**c**) gingival hypertrophy, pale pink gingiva, papillary and marginal gingival staining around 2.1 tooth.

**Figure 4:**
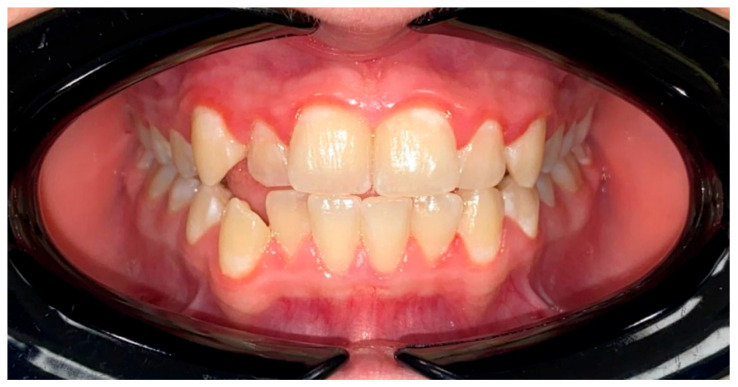
The intraoral photo protocol of a Group 2 patient. Gingival hyperemia is demonstrated. Occlusion in front.

Intraoral examination in ESKD adolescents revealed dense and pigmented dental plaque, calculus, and gingival hypertrophy of varying severity, but the gums were pale pink (Figure 3). Group 2 patients showed predominantly soft dental plaque and gingival hyperemia (Figure 4).

All periodontal indices were significantly higher in ESKD patients compared to the control (*p* < 0.0001). Group 1’s OHI-S averaged 1.93 ± 0.76, corresponding to an unsatisfactory individual oral hygiene, PMA averaged 31.97 ± 16.15%, indicating a moderate gingivitis, and PI averaged 2.32 ± 1.30, confirming a moderate periodontal disease (Figure 3). Group 2’s OHI-S averaged 1.01 ± 0.31, corresponding to a satisfactory individual oral hygiene, PMA averaged 15.20 ± 8.04%, and PI—1.04 ± 0.43 indicated mild gingivitis. Salivary pH in Group 1 was determined as slightly alkaline (7.34 ± 0.66), and in Group 2 as slightly acidic (6.27 ± 0.64) (*p* < 0.0001).

### 3.2. Oral Microflora in ESKD Adolescents

The G.A. Osipov chromatography–mass spectrometry results are shown in Table 3. Oral microbiota demonstrated opportunistic microbial contamination in Group 1 that differed from the control by the following species: *Bifidobacterium* spp., *Clostridium difficile*, *Nocardia asteroids*, *Rhodococcus* spp., *Staphylococcus aureus*, *Alcaligenes* spp., *Eggerthella lenta*, and *Candida* spp. Moreover, transient microorganisms such as *Pseudomonas aeruginosa*, *Klebsiella pneumoniae*, *Haemophilus parainfluenzae*, *Gemella morbillorum*, *Abiotrophy defectia*, *Herpes simplex*, etc., were found in Group 1.

According to classification by V.V. Khazanova et al. [22], oral microbiota disturbances were revealed in all ESKD adolescents. Grade 3 (grade I–II dysbiosis) was determined in 30.0% (*p* < 0.05), Grade 4 (II–III degree of dysbiosis) was determined in 46.7% (*p* < 0.0001), and Grade 5 (IV degree of dysbiosis) was determined in 23.3% of patients (*p* < 0.05). A total of 40.0% of Group 2 patients had no disturbances in oral microflora (*p* < 0.0001), Grade 2 (dysbiotic shift) was detected in 55.0% of Group 2 patients (*p* < 0.0001), and Grade 3 (I–II degree of dysbiosis) was determined in 5.0% (*p* < 0.05). Grades of oral dysbiosis in the studied groups are shown in Table 4.

Periodontopathogenic bacteria identified in the studied groups are shown in Table 5. *Tannerella forsythia*, the member of “red complex,” and *Aggregatibacter actinomycetemcomitans*, the member of “green complex,” were statistically significant. The frequency of *Tannerella forsythia* occurrence was 77% (*p* < 0.001) and the frequency of *Aggregatibacter actinomycetemcomitans* occurrence was 23% (*p* < 0.05). *Fusobacterium nucleatum*, belonging in the “orange complex,” was found in all patients (*p* > 0.05). *Prevotella intermedia*, *Treponema denticola*, and *Porphyromonas gingivalis* did not demonstrate statistically significant differences between the groups (*p* > 0.05).

### 3.3. Immunological Status

**Figure 5:**
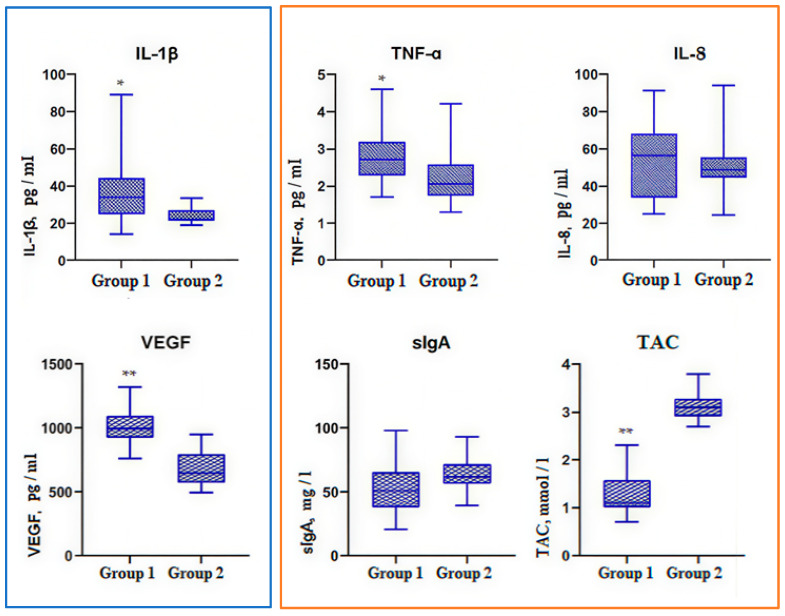
Salivary biomarkers in the studied groups. Note: Interleukin-1β (IL-1β); tumor necrosis factor alpha (TNF-α); interleukin-8 (IL-8); vascular endothelial growth factor (VEGF); secretory immunoglobulin A (sIgA); total antioxidant capacity (TAC); Group 1 (n = 30)—adolescents with end-stage chronic kidney disease; Group 2 (n = 20)—adolescents without renal pathology; IL-1β, VEGF—Welch’s *t*-test; TNF-α, IL-8, sIgA, TAC—Mann–Whitney U test; *—*p* < 0.05; **—*p* < 0.0001.

**Figure 6:**
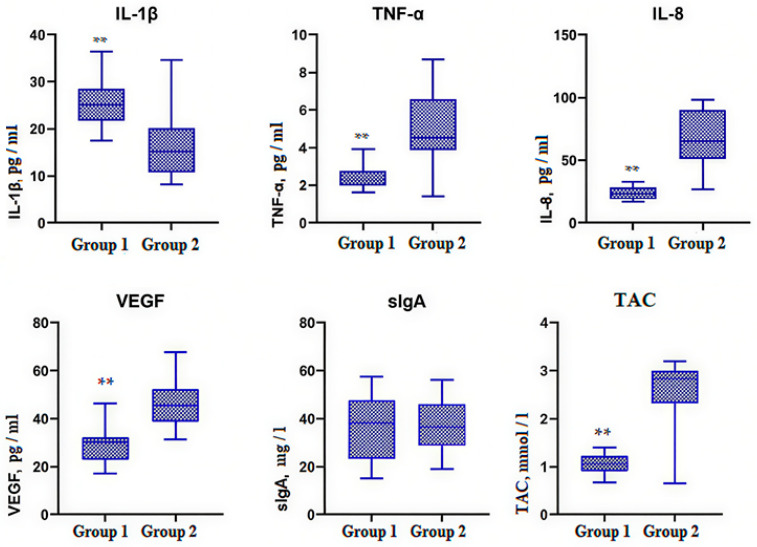
Gingival crevicular fluid biomarkers in the studied groups. Note: Interleukin-1β (IL-1β); tumor necrosis factor alpha (TNF-α); interleukin-8 (IL-8); vascular endothelial growth factor (VEGF); secretory immunoglobulin A (sIgA); total antioxidant capacity (TAC); Group 1 (n = 30)—adolescents with end-stage chronic kidney disease; Group 2 (n = 20)—adolescents without kidney pathology; Mann–Whitney U test; **—*p* < 0.0001.

The concentration of biomarkers was determined in two biological fluids: saliva (Figure 5) and GCF (Figure 6). IL-1β was higher in Group 1 compared to Group 2 both in saliva (*p* < 0.05) and in GCF (*p* < 0.0001). Salivary IL-1β was 33.58 (24.76–44.28) pg/mL in Group 1 and 22.73 (21.11–26.90) pg/mL in Group 2. IL-1β in GCF was 25.59 ± 4.83 pg/mL and 16.17 ± 6.68 pg/mL, respectively.

Salivary TNF-α in Group 1 (2.75 ± 0.66 pg/mL) was slightly higher than in Group 2, where it was 2.25 ± 0.80 pg/mL (*p* < 0.05), while TNF-α in GCF in Group 1 (2.39 ± 0.52 pg/mL) was two times lower than in Group 2 (4.86 ± 1.90 pg/mL) (*p* < 0.0001).

Salivary IL-8 was 55.58 ± 20.85 pg/ml in Group 1 and 51.28 ± 16.43 pg/mL in Group 2, so it did not demonstrate statistically significant differences between the groups. IL-8 in GCF in ESKD patients (23.85 ± 5.15 pg/mL) was significantly lower compared to the control, where IL-8 in GCF was 65.25 ± 23.48 (*p* < 0.0001).

Salivary VEGF in ESKD adolescents was 988.9 (898.2–1089) pg/mL, so significantly higher than in the control, where salivary VEGF was 650.9 (570–798.2) pg/mL (*p* < 0.0001). Exactly opposite VEGF values were found in GCF: VEGF in Group 1 (28.54 ± 6.85 pg/mL) was lower than VEGF in Group 2 (45.92 ± 9.09 pg/mL) (*p* < 0.0001).

Salivary sIgA in Group 1 was 53.80 ± 20.94 mg/L and in Group 2 63.13 ± 13.93 mg/L. sIgA in GCF was 36.67 ± 13.56 mg/L and 36.94 ± 11.17 mg/L in the studied groups, respectively. So, sIgA did not differ in either saliva or GCF (*p* > 0.05).

Group 1 TAC was 1.45 ± 0.51 mmol/L in saliva and 1.05 ± 0.22 mmol/L in GCF. Group 2 TAC was 3.14 ± 0.28 mmol/L in saliva and 2.42 ± 0.88 mmol/L in GCF. Thus, TAC in both studied fluids was significantly higher in healthy controls (*p* < 0.0001).

### 3.4. Correlation Analysis of Oral Microbiota and Biomarkers

As follows from Table 6, a statistically significant correlation was found between oral microbiota changes and salivary VEGF (r = +0.543; *p* < 0.001), salivary TNF-α (r = +0.418; *p* = 0.003), and salivary IL-1β (r = +0.365; *p* = 0.009). The identified relationship had moderate strength and reflected a simultaneous oral dysbiosis and the increasing concentration of the above-mentioned markers. Meanwhile, a strong inverse correlation was found between oral microbiota changes and salivary TAC (r = −0.753; *p* < 0.001). However, no statistically significant relationship was found between oral microbiota changes and salivary IL-8 and sIgA.

Oral microbiota changes had a statistically significant inverse medium strength correlation between almost all GCF markers, including VEGF (r = −0.670; *p* < 0.001), TNF-α (r = −0.523; *p* < 0.001), IL-8 (r = −0.632; *p* < 0.001), and TAC (r = −0.384; *p* = 0.006). The exceptions were IL-1β, which had a direct medium strength correlation (r = +0.327; *p* = 0.021), and sIgA, which did not have a statistically significant relationship with either periodontopathogens or oral microbial composition.

Correlation analysis of periodontopathogenic bacteria determined that *Fusobacterium nucleatum* had no association with any marker in either saliva or GCF, since this pathogen was detected in all studied patients.

Statistically significant direct correlation was found between *Tannerella forsythia* and salivary VEGF (r = +0.389; *p* = 0.006) and IL-1β, both in saliva (r = +0.445; *p* = 0.001) and in GCF (r = +0.347; *p* = 0.013). A moderate inverse relationship was found with VEGF (r = −0.574; *p* < 0.001), TNF-α (r = −0.537; *p* < 0.001), IL-8 (r = −0.600; *p* < 0.001), and TAC (r = −0.578; *p* < 0.001) in GCF.

*Treponema denticola* was statistically significantly associated only with a VEGF decrease in GCF (r = −0.295; *p* = 0.038).

A statistically significant direct medium-strength relationship was revealed between *Aggregatibacter actinomycetemcomitans* and salivary VEGF (r = +0.358; *p* = 0.011) and salivary IL-1β (r = +0.407; *p* = 0.003).

*Prevotella intermedia* was not associated with changes in any of the studied markers in either saliva or GCF.

*Porphyromonas gingivalis* was associated with increased salivary TNF-α (r = +0.478; *p* < 0.001) and IL-1β in both saliva (r = +0.315; *p* = 0.026) and GCF (r = +0.299; *p* = 0.035).

## 4. Discussion

The present study comprehensively investigated intraoral findings associated with ESKD in adolescents. The DMFT score was a bit lower in Group 1 compared to Group 2, but not statistically significant, whereas OHI-S, PMA, PI, and pH scores were higher in Group 1, and these differences were statistically significant. The incidence of dental caries in ESKD children varied greatly in the reviewed literature: some authors found a higher prevalence of caries in ESKD children compared to the control [24,25], while others found it lower [26,27], and A. Beyer et al. did not find a statistically significant difference in DMFT index in ESKD children and their peers without kidney pathology [28]. Such differences in data could be due to both factors predisposing to caries (vitamin D deficiency, poor oral hygiene, xerostomia, high-carbohydrate diet, etc.) and factors preventing caries (alkaline salivary pH and changes in the oral microbiota [29]).

Our study results showed a significant increase in periodontal indices in ESKD patients compared to the control. Moreover, both groups’ values exceeded normal dental index values [30,31]. Group 1’s OHI-S corresponded to an unsatisfactory individual oral hygiene, its PMA indicated moderate gingivitis, and its PI confirmed a moderate periodontal disease. It was consistent with the results cited by B. Sezer et al. and T.M.C. Silva et al. [32,33]. The scoping review showed that ESKD patients had more dental plaque and gingival inflammation than the control [4]. Healthy children had limited or no dental calculus, while CKD adolescents more often had it due to increased oral urea and more alkaline salivary pH compared to the control [9]. In addition, it is known that xerostomia is one of the periodontal disease risk factors in ESKD adolescents because of decreased salivation and, as a result, there is no natural dental self-cleaning [4]. Increased PI values in Group 1 were based on immunosuppressive DIGO [34].

It is important to note that in our study, OHI-S and PMA exceeded normal values in patients without renal pathology, who also had dental plaque and mild gingivitis. Individual oral hygiene in most adolescents is insufficient and requires training, dynamic follow-up, and motivation. However, Group 1’s intraoral examination, despite the abundance of dental plaque and gingival hypertrophy, discovered pale pink gums compared to Group 2, who had gingival hyperemia.

ESKD patients experience changes in saliva (increased urea and ammonia levels, elevated pH), changes in oral mucosa and gums, and poor oral hygiene [35]. These changes can lead to oral dysbiosis.

The oral cavity is a complex ecosystem. Many microorganisms listed in Table 3 are commensals. Their role shifts from harmless to pathogenic when the microbial balance is disrupted, the oral environment changes, or the host’s immune defense is compromised [36]. Our study demonstrated oral microbiota changes in both groups of patients (Table 4). Elevated levels of *Bifidobacteria* can potentially lead to dental caries [37]. *Clostridium difficile* is frequently found in dysbiotic subgingival biofilm in conditions of poor oral hygiene and periodontal diseases [38]. The co-occurrence relationship between *Actinomyces* spp. and *Eggerthella lenta* plays one of the key roles in causing periodontal inflammation [39]. *Nocardia* spp., *Rhodococcus* spp., *Alcaligenes* spp., and *Candida* spp. tend to affect immunocompromised hosts [40,41]. The oral cavity acts as a reservoir for *Staphylococcus aureus* [42] and its oral carriage has most frequently been associated with decreased salivary secretion, with carious lesions and periodontal diseases [43].

More than half of healthy adolescents in our study showed Grade 2 of dysbiosis, while ESKD adolescents showed more severe grades of dysbiosis (Table 5). It was consistent with the results cited by J. Hu et al., who found that there was a correlation between a decrease in glomerular filtration rate and decreased numbers of commensal genera such as *Veillonella*, *Streptococcus*, and *Alloscardovia*, as well as increased numbers of pathogenic species such as *N. gonorrhoeae*, *N. meningitides*, *Streptococcus sanguinis*, *Streptococcus mutans,* etc. [11]. ESKD patients are observed with increased numbers of *Enterobacteriaceae*, *Actinetobacter* spp., and *Vibrio* spp. in cavitiesavity, which indicates the dysbiosis. *Klebsiella pneumoniae* and *Pseudomonas* spp. are pathogenic microorganisms and often associated with post-transplant infections [12]. *Haemophilus parainfluenzae*, *Gemella morbillorum*, and *Abiotrophia defectiva* are underestimated reasons for common infectious complications, such as bacterial endocarditis and endarteritis [9]. Moreover, oral microbiota is significantly affected by immunosuppressive drugs used to prevent graft rejection, especially in the first months after transplantation, when the drug dosages are high [44].

Among the identified periodontopathogenic bacteria, *Fusobacterium nucleatum* was found in all patients. *Fusobacterium nucleatum* is an opportunistic dental plaque bacterium, but the combined effects of a dysbiotic microbial community and an impaired immune response ultimately cause periodontal disease [45]. In addition, *Fusobacterium nucleatum*, due to its metabolic capabilities, creates a favorable environment for the growth of other periodontopathogenic bacteria, such as *Porphyromonas gingivalis* [46]. *Tannerella forsythia* and *Aggregatibacter actinomycetemcomitans* in the subgingival plaque were statistically significant in our study (Table 3). A modern review shows that periodontopathogenic bacteria have been found to be more prevalent in CKD patients than in the general population [47], which is consistent with our study results.

Our biochemical analysis showed that salivary IL-1β, TNF-α, and VEGF were el-evated in Group 1 compared to Group 2; meanwhile, in GCF, only IL-1β was higher in ESKD children than in the control. TNF-α, IL-8, and VEGF in GCF were statistically significantly reduced in ESKD patients compared to the control. TNF-α and IL-8 decrease might be associated with their inhibition when taking immunosuppressive drugs [48], and VEGF decrease could be due to immunosuppression block of the VEGF/VEGFR signaling pathway, which hinders angiogenesis in the gum [17]. sIgA in GCF did not differ in the studied groups of adolescents, and sIgA in saliva was statistically insignificantly lower in Group 1. H.M. Sylenko et al. confirmed that chronic periodontal inflammation was accompanied by salivary sIgA decrease and recommended immunomodulatory drugs in complex periodontal treatment [49]. We found that TAC in both biological fluids in ESKD adolescents was two times lower than in patients without kidney pathology. It might be due to oxidative stress increased by immunosuppression [19].

A statistically significant correlation was found between oral microbiota changes and salivary VEGF, salivary TNF-α, and salivary IL-1β. These correlations suggest that oral dysbiosis might lead to the high level of these biomarkers. Other authors confirmed the relationship between oral microbiota disruption and salivary biomarkers [50]. At the same time, a strong inverse correlation was found between oral microbiota composition and TAC. Indeed, oxidative stress develops during oral dysbiosis, so antioxidant activity is reduced [51].

N.A. Hickey et al. suggested that CKD correlated with chronic periodontitis development, since pH changes and gingival hyperplasia detected in CKD patients could create favorable environment for periodontopathogenic bacteria growth [35], and systemic inflammation in chronic periodontitis could aggravate CKD due to oxidative stress and cytokine production [52]. It is consistent with our study results.

Correlation analysis of periodontopathogenic bacteria determined that *Fusobac-terium nucleatum* had no association with any marker in either saliva or GCF, since this pathogen was detected in all studied patients.

Statistically significant direct correlation was found between *Tannerella forsythia* and salivary VEGF and IL-1β both in saliva and in GCF. A moderate inverse correlation was found between *Tannerella forsythia* and VEGF, TNF-α, IL-8, and TAC in GCF. *Porphyromonas gingivalis* was associated with increased salivary TNF-α and IL-1β in both saliva and GCF. *Treponema denticola* was statistically significantly associated only with a VEGF decrease in GCF (*p* < 0.05). A statistically significant direct medium-strength relationship was revealed between *Aggregatibacter actinomycetemcomitans* and salivary VEGF and salivary IL-1β. The studied literature most often shows a direct correlation between periodontopathogenic bacteria and increased levels of proinflammatory cytokines. H. Lu et al., J. Mahendra et al., and M. Mazurek-Mochol, in their studies, confirmed a relationship between the levels of IL-8, TNF-α, VEGF, and periodontopathogenic bacteria, thereby demonstrating the role of inflammatory biomarkers in periodontal diseases and systemic inflammation contribution in CKD [48,53,54]. J.M. Almerich-Silla et al.’s study proved that oxidative stress parameters correlated with some periodontopathogenic bacteria, including *Tannerella forsythia* [55], which was consistent with our results identifying a medium-strength inverse relationship between *Tannerella forsythia* and TAC in GCF.

The difference in salivary and GCF biomarkers might be associated with the fact that both innate and adaptive immunity impaired in CKD due to prolonged periodontal inflammation. Lower cytokine synthesis in response to periodontopathogenic bacteria could be presumably due to dialysate endotoxins, dysfunction of antigen-presenting cells, and immunosuppression of macrophages [47].

Correlations between studied biomarkers and periodontopathogenic bacteria in combination with a significant increase in periodontal indices might help to demonstrate periodontal inflammation in ESKD adolescents, despite the pale pink gums compared to Group 2, who had gingival hyperemia. This fact confirms subclinical inflammatory process and molecular diagnostics as necessity to oral health assessment in ESKD adolescents.

Our study had some limitations that should be acknowledged. The sample size was limited to the number of patients admitted to the hospital during the study period by the diagnostic cut-off at a single follow-up point. Therefore, it was a pilot study. The paucity of preliminary investigations addressing this subject further restricted opportunities to refine both the study design and methodology. One dental examiner may have resulted in measurement variability. Participants were not matched for socioeconomic status and diet, so it could act as a confounding factor affecting the observed results. The DMFT index and caries-related results were not significant in our study, so it might be better to try any other indices such as ICDAS, DDE, etc. We acknowledge that our findings should be interpreted as preliminary. Additional longitudinal studies on larger samples with different designs are needed to obtain more reliable conclusions.

## 5. Conclusions

Comprehensive oral assessment showed that ESKD adolescents had poor oral hygiene, gingival hypertrophy, dental calculus, and significant oral dysbiosis including periodontopathogenic bacteria. Evaluation of biomarkers in saliva and GCF allowed us to vindicate inflammation, dysbiosis severity, and periodontal diseases, and also demonstrated the influence of immunosuppressive treatment during ESKD on the local immune response. Preliminary findings of the study may highlight the significance of interdisciplinary collaboration between dental and medical practitioners in the evaluation and management of oral health among individuals undergoing ESKD.

## Figures and Tables

**Table 1:** Baseline characteristics of the study groups.

Variable		Groups		*p*-Value
		Group 1 (n = 30)	Group 2 (n = 20)	
Age, mean ± SD (years)		13.4 ± 1.8	13.7 ± 1.9	0.58 ^1^
Median age (years)		13	13	–
Age range (min–max), years		12–17	12–17	–
Female, n (%)		9 (30%)	4 (20%)	0.52 ^2^
Toothbrushing frequency, n (%)	Once a day	19 (63.3%)	13 (65.0%)	1.000 ^2^
	Twice or more	11 (36.7%)	7 (35.0%)	
Dental flossing using, n (%)	No	27 (90.0%)	18 (90.0%)	1.000 ^2^
	Yes	3 (10.0%)	2 (10.0%)	

Note: Group 1 (n = 30)—adolescents with end-stage chronic kidney disease, Group 2 (n = 20)—adolescents without urinary pathology. Mean ± Standard Deviation (mean ± SD) value. ^1^—Welch’s *t*-test. ^2^—Fisher’s exact test.

**Table 2:** Oral health assessment of the patients.

Variable	Groups		*p*-Value
	Group 1 (n = 30)	Group 2 (n = 20)	
DMFT	1.27 ± 2.12	1.6 ± 1.45	>0.05
OHI-S	1.93 ± 0.76	1.01 ± 0.31	<0.0001
PMA, %	31.97 ± 16.15	15.20 ± 8.04	<0.0001
PI	2.32 ± 1.30	1.04 ± 0.43	<0.0001
pH	7.34 ± 0.66	6.27 ± 0.64	<0.0001

Note: Group 1 (n = 30)—adolescents with end-stage chronic kidney disease, Group 2 (n = 20)—adolescents without urinary pathology. DMFT—decayed, missing, filled teeth index; OHI-S—J.C. Greene and J.R. Vermillion oral hygiene index-simplified; PMA—papillary marginal attached index; PI—A.L. Russell periodontal index. Mean ± Standard Deviation (Mean ± SD) values. Student’s *t*-test.

**Table 3:** Opportunistic oral pathogens in the studied groups.

Pathogen	Groups		*p*-Value
	Group 1 (n = 30)	Group 2 (n = 20)	
*Actinomyces* spp. (median × 10^5^), CFU/mL	451	100	>0.05
*Bifidobacterium* spp. (median × 10^5^), CFU/mL	1539	710	<0.05 *
*Clostridium difficile* (median × 10^5^), CFU/mL	355	204	<0.05 *
*Eubacterium* spp. (median × 10^5^), CFU/mL	8693	4935	>0.05
*Nocardia asteroids* (median × 10^5^), CFU/mL	1943	184	<0.05 *
*Pseudonocardia* spp. (median × 10^5^), CFU/mL	379	76	>0.05
*Rhodococcus* spp. (median × 10^5^), CFU/mL	976	521	<0.05 *
*Staphylococcus aureus* (median × 10^5^), CFU/mL	1445	401	<0.05 *
*Streptococcus mutans* (median × 10^5^), CFU/mL	1465	1038	>0.05
*Alcaligenes* spp. (median × 10^5^), CFU/mL	352	43	<0.05 *
*Streptococcus pneumonia* (median × 10^5^), CFU/mL	189	70	>0.05
*Candida* spp. (median × 10^5^), CFU/mL	5492	1864	<0.05 *
*Aspergillus* spp. (median × 10^5^), CFU/mL	4484	3324	>0.05
*Eggerthella ribbon* (median × 10^5^), CFU/mL	1160	440	<0.05 *
*Ruminococcus* spp. (median × 10^5^), CFU/mL	521	370	>0.05
*Propionibacterium freudenreichii* (median × 10^5^), CFU/mL	2378	1847	>0.05

Note: Group 1 (n = 30)—adolescents with end-stage chronic kidney disease, Group 2 (n = 20)—adolescents without renal pathology. CFU—colony-forming units. Mann–Whitney U test; *—*p* < 0.05.

**Table 4:** Grades of oral dysbiosis in the studied groups.

Grade	Groups		*p*-Value
	Group 1 (n = 30)	Group 2 (n = 20)	
Grade 1, n (%)	0 (0.0%)	8 (40.0%)	<0.0001 **
Grade 2, n (%)	0 (0.0%)	11 (55.0%)	<0.0001 **
Grade 3, n (%)	9 (30.0%)	1 (5.0%)	<0.05 *
Grade 4, n (%)	14 (46.7%)	0 (0.0%)	<0.0001 **
Grade 5, n (%)	7 (23.3%)	0 (0.0%)	<0.05 *

Note: Group 1 (n = 30)—adolescents with end-stage chronic kidney disease, Group 2 (n = 20)—adolescents without renal pathology. Grade 1—normal state of oral microbiota; Grade 2 (dysbiotic shift)—predominance of a single opportunistic microorganism with preservation of the normal species composition of the oral microbiota; Grade 3 (grade I–II dysbiosis)—detection of 2–3 pathogenic species accompanied by a partial reduction in normal microbiota; Grade 4 (grade II–III dysbiosis)—detection of a pathogenic monoculture with a marked decrease or complete absence of representatives of the normal microbiota; Grade 5 (grade IV dysbiosis)—the presence of associations of pathogenic bacterial species with a marked increase in yeast-like fungi. Fisher’s exact test; *—*p* < 0.05, **—*p* < 0.001.

**Table 5:** Frequency of periodontopathogenic bacteria occurrence in the studied groups.

Periodontopathogenic Bacteria	Groups		*p*-Value
	Group 1 (n = 30)	Group 2 (n = 20)	
*Aggregatibacter actinomycetemcomitans*, %	23	-	<0.05 *
*Fusobacterium nucleatum*, %	100	100	>0.05
*Prevotella intermedia*, %	13	-	>0.05
*Treponema denticola*, %	10	-	>0.05
*Tannerella forsythia*, %	77	-	<0.001 **
*Porphyromonas gingivalis*, %	10	-	>0.05

Note: Group 1 (n = 30)—adolescents with end-stage chronic kidney disease, Group 2 (n = 20)—adolescents without renal pathology. Color-coding of the bacterial groups was based on their association with microbial complexes by Socransky et al. [9]. Fisher’s exact test; *—*p* < 0.05, **—*p* < 0.001.

**Table 6:** Correlation between biomarkers, periodontopathogenic bacteria, and oral microbiota composition.

Biomarker		Correlation Coefficient Value *						
		*Fusobacterium* *nucleatum*	*Tannerella forsythia*	*Treponema denticola*	*Aggregatibacter* *actinomycetemcomitans*	*Prevotella intermedia*	*Porphyromonas* *gingivalis*	Oral Microbiota Composition
**Saliva**	VEGF, pg/mL	—	+0.380 **	−0.066	+0.358 **	+0.169	+0.167	+0.543 **
	TNF-α, pg/mL	—	+0.242	−0.104	+0.052	−0.018	+0.478 **	+0.418 **
	IL-1β, pg/mL	—	+0.445 **	−0.159	+0.407 **	−0.109	+0.315 **	+0.365 **
	IL-8, pg/mL	—	+0.256	−0.110	+0.216	−0.075	+0.124	+0.210
	sIgA, mg/L	—	−0.064	−0.184	−0.029	+0.220	−0.093	−0.249
	TAC, mmol/L	—	−0.658 **	−0.237	−0.245	−0.262	−0.165	−0.753 **
**GCF**	VEGF, pg/mL	—	−0.574 **	−0.295 **	−0.153	−0.054	−0.140	−0.670 **
	TNF-α, pg/mL	—	−0.537 **	−0.112	−0.249	−0.125	−0.125	−0.523 **
	IL-1β, pg/mL	—	+0.347 **	+0.097	+0.139	+0.136	+0.299 **	+0.327 **
	IL-8, pg/mL	—	−0.600 **	−0.165	−0.267	−0.177	−0.123	−0.632 **
	sIgA, mg/L	—	−0.097	−0.167	+0.126	+0.209	−0.055	+0.013
	TAC, mmol/L	—	−0.578 **	−0.180	−0.243	−0.193	−0.171	−0.384 **

Note: Gingival crevicular fluid (GCF); interleukin-1β (IL-1β); tumor necrosis factor alpha (TNF-α); interleukin-8 (IL-8); vascular endothelial growth factor (VEGF); secretory immunoglobulin A (sIgA); total antioxidant capacity (TAC); Group 1 (n = 30)—adolescents with end-stage chronic kidney disease; Group 2 (n = 20)—adolescents without renal pathology; *—the relationship between biomarkers and periodontopathogenic bacteria was assessed using Pearson point-biserial correlation coefficient, and the relationship between biomarkers and oral microbiota composition was assessed using Spearman correlation coefficient. **—statistically significant relationship (*p* < 0.05). Color-coded gradations characterize the strength of the identified correlation.

## Data Availability

Data are unavailable due to privacy or ethical restrictions.

## Abbreviations

The following abbreviations are used in this manuscript:

CKD	Chronic kidney
ESKD	End-stage chronic kidney disease
GCF	Gingival crevicular fluid
IL-1β	Interleukin-1β
IL-8	Interleukin-8
TNF-α	Tumor necrosis factor alpha
VEGF	Vascular endothelial growth factor
sIgA	Secretory immunoglobulin A
TAC	Total antioxidant capacity
DMFT	Decayed, missed, filled teeth index
OHI-S	Oral hygiene index-simplified
PMA	Papillary marginal attached index
PI	Periodontal index
PCR	Polymerase chain reaction
ELISA	Enzyme-linked immunosorbent assay
